# Monocular Robust Depth Estimation Vision System for Robotic Tasks Interventions in Metallic Targets †

**DOI:** 10.3390/s19143220

**Published:** 2019-07-22

**Authors:** Carlos Veiga Almagro, Mario Di Castro, Giacomo Lunghi, Raúl Marín Prades, Pedro José Sanz Valero, Manuel Ferre Pérez, Alessandro Masi

**Affiliations:** 1 CERN, EN-SMM Survey, Measurement and Mechatronics group, 1217 Geneva, Switzerland; 2 Centro de Automatica y Robotica (CAR) UPM-CSIC, Universidad Politecnica de Madrid, 28006 Madrid, Spain; 3 Interactive Robotic Systems Lab, Jaume I University of Castellón, 12006 Castellón de la Plana, Spain

**Keywords:** vision, robotic interventions, eye-in-hand, tracking, hazardous environments, radioactive scenarios, human-supervisory control, telerobotics

## Abstract

Robotic interventions in hazardous scenarios need to pay special attention to safety, as in most cases it is necessary to have an expert operator in the loop. Moreover, the use of a multi-modal Human-Robot Interface allows the user to interact with the robot using manual control in critical steps, as well as semi-autonomous behaviours in more secure scenarios, by using, for example, object tracking and recognition techniques. This paper describes a novel vision system to track and estimate the depth of metallic targets for robotic interventions. The system has been designed for on-hand monocular cameras, focusing on solving lack of visibility and partial occlusions. This solution has been validated during real interventions at the Centre for Nuclear Research (CERN) accelerator facilities, achieving 95% success in autonomous mode and 100% in a supervised manner. The system increases the safety and efficiency of the robotic operations, reducing the cognitive fatigue of the operator during non-critical mission phases. The integration of such an assistance system is especially important when facing complex (or repetitive) tasks, in order to reduce the work load and accumulated stress of the operator, enhancing the performance and safety of the mission.

## 1. Introduction

Maintenance of equipment in scientific research organisations, like the European Centre for Nuclear Research (CERN), is critical in order to ensure the correct operation of the experimental infrastructure. However, people’s access to experimental facilities is not always possible due to their hazardous characteristics such as the presence of radiation, high magnetic field and possible lack of oxygen in the context of underground areas. Telerobotic platforms can perform some of the maintenance tasks in a safer and more reliable manner. As a matter of fact, up until now, the CERNBot robotic platform [1] has been used in more than one hundred real interventions, which have been very successful, and has enabled the accumulation of experience in order to improve future works.

Besides this, it is important to take into consideration the fact that, due to the huge amount of equipment to be maintained, the operations can be repetitive. As an example, in the Large Hadron Collider (LHC) accelerator there are around 4500 BLM sensors, which have to be checked regularly to assure the good performance of the system. In these situations, the use of standard and semi-autonomous automation techniques can help with designing accurate and safe robotic systems to perform the interventions [2,3]. Moreover, other kinds of necessities exist, such as recovering radioactive targets, which need a great level of expertise on both the robot manual operation and the scientific machine. In these scenarios, the presence of unexpected situations might come up, such as obstacles (e.g., cables), magnetic fields and radiation that might produce communication loss, among others.

Considering such necessities, it is important that the operator is able to interact with the remote robotic systems in a multimodal way, being able, for example, to launch high-level semi-autonomous behaviours when the tasks are repetitive and safe (e.g., inspection for creating radiation maps) and also permitting the control of the robots at low level (e.g., master-slave bilateral control with force feedback). The use of such a multimodal human-supervisory telerobotic system helps enormously in order to avoid the operator’s cognitive fatigue [1,4].

In fact, the use of a human-supervisory control system enables the ability to always have a human expert in the loop, so as to achieve the required safety measures.

**Figure 1:**
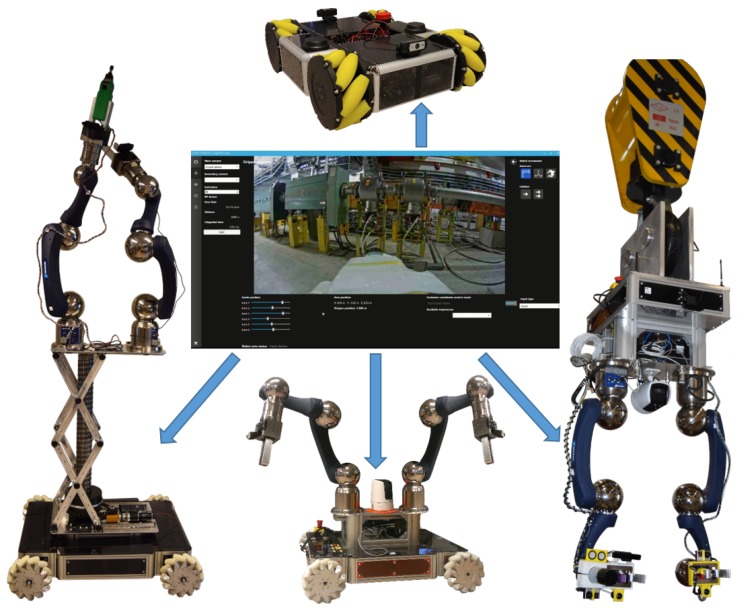
Unified multimodal human–robot interaction (HRI) to control the modular and reconfigurable CERNBot robotic system.

The success of the operations has been possible thanks to the modularity of the system, which permits the adapting of the mechanical design and software architecture to the specific mission plan. For this, the CERNBot modular robot has been designed [5] (see Figure 1). The robot can be easily adapted by changing the tools, the position, number of arms and wheels, among others. In addition, the server software architecture (i.e., CERN Robotic Framework) and the user interface adapt dynamically to the current robot configuration, using a multimodal and unified human–robot interaction (HRI) [6].

According to the acquired experience on robotic interventions and taking into account their growing complexity, further steps need to be performed in order to guarantee safety and efficiency. For this, reliable computer vision, object recognition and grasping modules need to be studied and integrated in the system, providing the expert operator with more sophisticated tools in order to reach the expected quality during operation, while also increasing safety and accuracy. The computer system also needs to be rapidly configured and installed, so the context of this paper focuses on the use of monocular cameras for on-hand robotic vision control, which can be easily installed in specific places of the gripper or tools. Moreover, the vision system needs to be reliable under occlusions, reflections and on metallic surfaces with lack of features, also providing specific added-values such as the automatic calculations of depth. These are in fact the main goals of the vision system described in this paper.

### 1.1. State of the Art

In the scientific literature a great pool of computer vision systems providing the position and orientation of targets with respect to the current camera situation can be found. Some of these systems incorporate robotic actuators with an on-hand 2D camera [7], attached in conjunction with sensors such as a laser [8,9], or a sonar [10]. Others are based on the use of single monocular cameras [11]. Different hardware setups can be used according to the issue to be addressed, such as the installation of cameras on the mobile robot, or on the scene (in a fixed position) [12,13], in order to provide an environmental third view, using also eye-in-hand techniques [14,15].

Sensor fusion systems can make use of cameras with depth information like Kinect [16] and RealSense [17]. Additionally, some related works make use of stereo cameras to track a target, in which both cameras are placed at a predetermined distance and rotation, calculating a whole 3D reconstruction of the scene [18,19,20] using Epipolar Geometry [21]. A single-camera system can also be deployed [22] simulating a stereo system, either using markers [23], or previously establishing the separation parameters among two images [24], knowing the relationship between the key-points of both images, necessary to build the Epipolar Geometry. Nobakht and Liu [25] proposed a method to estimate the position of the camera with respect to the world, using a known object to compute the position by Epipolar Geometry.

Although time-of-flight (ToF) cameras [26] procure a set of key-points (based upon low modulation infrared light (20 MHz)) for a 3D reconstruction by the camera intrinsic parameters, they depend largely on the object material.

All the systems presented above lead to a significant growth in the robotic platform hardware, for which the accuracy depends to a great extent on the environmental light, on the reflection against the target, and on the material. Only a few have faced the problem to recognise metallic objects, giving some promising results by the use of neural networks, while still presenting errors above 10% [27].

The present paper provides an step forward in order to allow a remotely supervised robotic system to recognise, track and estimate the position of metallic targets in real industrial and scientific scenarios. Results are very promising, which have been tested and validated in real interventions in the CERN tunnel facilities, during maintenance operations.

### 1.2. Problem Formulation

According to the current state of the art and the real necessities to be solved in robotic operations at CERN, to the best of the authors’ knowledge, there is no vision-based system that allows reliable object recognition and tracking of metallic targets in scenarios with partial occlusions, reflections and luminosity constraints, permitting also the calculation of distances to the object using a simple monocular camera, which can be installed in a specific position of the gripper. As a matter of fact, for further experimentation and user operator efficiency, it is also necessary to provide a grasping determination module, which can approach and guide the robot to the target in a simple and safe manner.

Moreover, considering the necessity to use such an Real-Time (RT) tracking vision system for both the operator’s feedback and the robot arm control to assist teleoperation, we deemed fit to focus on these techniques, which provide the robot position and orientation in relation to an image pattern taken from the scene and optimising them by adding the utility of computing the depth information of metallic objects (e.g., screws, connectors, etc.), focusing on the robustness and efficiency of the algorithm. As stated in the results, the integration of such tracking techniques have already been done and validated within the structure of the CRF (CERN Robotic Framework), with the purpose of using them in the interventions that are currently being carried out. With that in mind, the proposed solution contained in this paper exploits the transformation matrix of the robot to localise the current camera position (eye-in-hand configuration) and to determine the distance to the target (i.e., depth estimation). After the integration of the system in the CERN’s HRI [28] (see Figure 1), this can trigger the tracking and depth estimation for any of the objects present in the scene.

The vision system also includes an object recognition module (deep neural network-based), which does not only accept the Region of Interest (ROI) input over an unknown target selected manually by the operator but also searches autonomously for the objects, letting the operator interact with the robot by referring to objects instead of bounding boxes, and enabling further experimentation to carry out semi-autonomous tasks on the recognised metallic pieces (e.g., motion planning and grasping execution).

It is very important that the vision system works at a high performance, since most remote operations rely on real time visual feedback to the operator, although the 100% real-time capability cannot be really fulfilled because the 4G network does not provide such feature. This information is the main link between the human-expert and the robot, which is being operated remotely. The visual feedback needs to be provided to the user at a minimum delay, in order to avoid move-and-wait human-robot interactions, which would affect the efficiency of the system. Thus, when the teleoperation is carried out, the visual feedback has to work concurrently with the estimation and guidance process, which provide information that can be represented in the user interface using augmented reality techniques. On the other hand, for the autonomous and semi-autonomous tasks that do not require the RT feedback to the user, it is possible to gather visual data for further analysis.

In summary, this paper presents a novel solution to extend the capabilities of a supervised HRI in order to improve the guidance of a robotic arm (see Section 4). For this, the system includes a novel depth estimation solution, as well as a deep learning-based Faster-Regions with Convolutional Neural Network (RCNN) Features model [29] with Resnet-101 object recognition, which work efficiently in metallic surfaces, having unexpected reflections, partial occlusions and lack of visibility. Besides this, the vision system has been designed to work on board, without needing special hardware, also enabling the use of a broad range of monocular cameras in the market (e.g., black&white, endoscope and large full-High Definition (HD) Pan–Tilt–Zoom (PTZ) cameras), as can be seen in Table 1. This also enables the increase of the number of available cameras to the operator, facilitating the operation task.

## 2. Preliminary Experiments

As a preliminary step, in this section a comparative of different tracking algorithms using the above cameras and their calibration procedure is presented.

### 2.1. Tracking Algorithms Comparison

First of all, several tracking algorithms from the scientific literature were tested in order to better understand their performance in real robotic intervention conditions. The results can be summarised as follows:

- The Boosting algorithm [30] uses a set of techniques that mixes several weak classifiers algorithms to create a more robust solution. It showed the fastest performance when evaluating the features, while presenting a very low accuracy.
- Babenko et al. [31] present a robust object tracking Multiple Instance Learning (MIL)-based algorithm [32], which, although it was showing high precision, the computational time was higher too, due to the fact that it considers a set of training samples that can be ambiguous, as a single object can have many alternative instances that describe it.
- The Tracking-Learning-Detection (TLD) algorithm [33] tries to localise all the similarities within the scene. Thanks to this behaviour, it is capable of facing temporal occlusions, but it obtains a large number of miss-detections in scenarios with metallic parts, as well as higher computational time consumption.
- A version of the Kernelized Correlation Filters (KCF) algorithm [34] has been implemented. This algorithm, which is based on Histogram of Oriented Gradients (HOG) [35,36], has shown good computational performance and the greatest accuracy by tracking different kinds of objects. This is the algorithm that has been used as the basis for the solution implementation presented in this paper.

In fact, Hare et al. [37] compared their own tracking algorithm with the recently released ones, obtaining interesting results regarding those arising from HOG descriptors, instead of others derived from Haar-like features [38,39,40] such as Boosting, since HOG describes the object shape by way of edges detection or its distribution of intensity gradients after histograms concatenation from a set of small connected regions that were split from the main ROI. Then, in order to gain an improvement that is invariant to shadows and luminosity, it increases the accuracy, normalising these histograms.

Therefore, since our work is based on the tasks’ execution on metallic surfaces, the appearance of a large number of reflections can be readily detected, forcing us to dismiss the Haar-based algorithms as they are grounded in the pixel’s light intensity instead of HOG, which provides a solution more in accordance with our requirements.

### 2.2. Camera Calibration

The calibration of the set of cameras that were used in this project becomes a critical preliminary step in order to obtain the necessary parameters that allow a proper execution of the robotic task. The camera calibration can be performed in two steps, by calculating both the intrinsic and extrinsic parameters:

- *Intrinsic parameters*: The OpenCV solution [41] was used for this purpose, by applying the classical black-white chessboard, obtaining the distortion coefficient and the camera matrix (see Equation (1)).
  (1) $$CM=\left|\begin{array}{ccc} f_{x} & 0 & c_{x}\\ 0 & f_{y} & c_{y}\\ 0 & 0 & 1 \end{array}\right|$$
  Although the well-known distortion present in current pinhole cameras, this does not present an issue for the aim of this work, as it is possible to discard the distortion coefficient. However, the camera matrix provides the essential values for this aim, where $f_{x}$ and $f_{y}$ are the focal length in **X** and **Y** axis, respectively, and $c_{x}$ and $c_{y}$ are the optical centres expressed in pixels coordinates.
- *Extrinsic parameters*: Unlike the intrinsic parameters, this calibration provides the camera position and orientation in regards to the frame (i.e., the base of the robot). In Reference [42] a fast technique to carry out the task is presented, which is fully implemented in the ViSP library [43]. Due to the fact that the robotic system has been designed to be modular and easily re-configurable, including tools, actuators and sensors re-positioning, this calibration technique has been demonstrated to be very appropriate, due to the fact that the camera selection, as well as its position, changes assiduously (see Table 2).

Therefore, the estimation of the camera position in regards to the end-effector ($M_{e}^{c}$) is estimated by the Equation (2), which is linear least squares-based, making use of a set of the homogeneous transformation matrix of the robot ($M_{e}^{f}$) and the transformation matrix of the camera with respect to the pattern ($M_{o}^{c}$), both for each picture.

Finally, the transformation of the camera, with respect to the end-effector, is added to the robot matrix as the last joint of the robot configuration.

(2) $$M_{e}^{c}\to \left\{M_{e_{i}}^{f},M_{o_{i}}^{c}\right\}$$

## 3. System Overview

**Figure 2:**
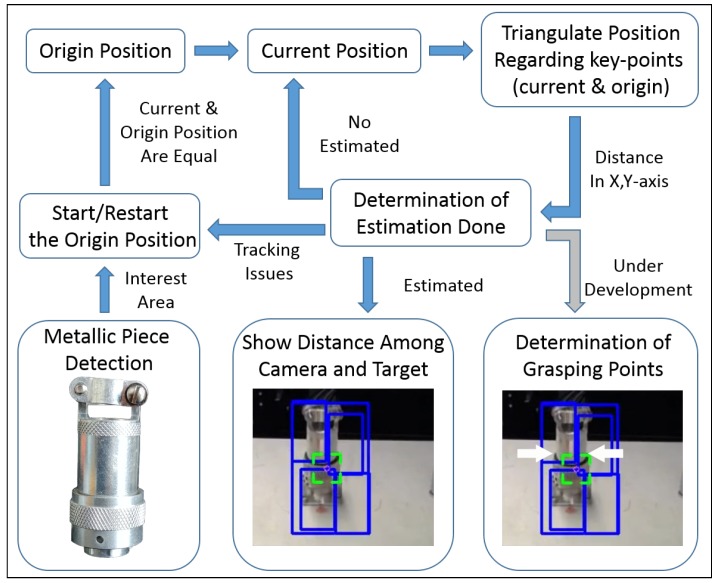
Diagram of the general principle of the system’ operation.

A new vision-based set of software engineering tools, consisting of a tracking algorithm, in conjunction with an object recognition module and depth estimation system, has been deployed to enhance the usability of a multimodal User Interface, which is in fact the user expert entry point to the system. The vision system acts as a server, listening for requests from the user interface, triggering the method shown in Figure 2.

The algorithm is based on tracking traces and computes the distance between any kind of mono-camera attached at the end-effector of the robotic arm (as shown in Table 1 and Table 2), to a selected metallic object with a lack of vision features, partially occult, or under reflections.

The procedure needs, as an input, a region of interest to be tracked. The ROI is extracted from the object recognition module and confirmed by the selection of the user. This ROI is split into four isolated and coordinated tracking areas (i.e., four trackers), which will depend on a parent one. Then it will lay a virtual set of key-points from the centre of each tracker and these key-points will be used as a correlation between the pair of images. The whole procedure shall be taken as a reference and replicated for every current frame, with the aim of triangulating the position of the target regardless of the movement performed on the *X* and *Y*-axis.

Taking advantage of the transformation matrix of the robot, once a pair of frames with different robot TCP positions have been analysed by the system, a normal distribution begins to be fed, with the purpose of deciding on the estimation of the target’s depth, looking for an error under a minimum pre-established experimental threshold (0.05% by default). If so, all the data (depth estimation, the percentage of error, and the distance between the ROI and the centre of the scene) shall be shown through the GUI by the AR module. If the system detects any tracking problems triggered by the implemented thresholds, or is taking a long time for such an estimation, it will restart the triangulation with a new reference.

It is important to clarify that current robot architecture uses three main computers: (1) the server on the robot where the cameras are connected, (2) the HRI operator computer and (3) the object recognition module computer where the neural network is being executed. In fact, the object recognition server is provided with a NVidia GTX1080 GPU and 32 GB of RAM, in order to improve its performance.

The tracking and depth estimation loop is going to be enhanced by a Grasping Determination module that is under development, enhancing previous experiments on fast 2D/3D grasping determination and execution. This module will allow the calculation of a list of stable grasping points that can be used by the operator to perform a picking task in a safe, accurate and supervised manner.

## 4. Target Tracking, Surrounding and Approach

One of the most important steps on the human-supervised telerobotic interventions is to track the target, maintain the camera’s focus on it and help the operator to bring the robot to an approached position in order to prepare the required interaction.

For that purpose, the computer vision system maintains the target in the field of view and assists the operator while approaching the target, in a remote controlled supervised manner.

The main difficulties to be solved in order to accomplish the task are the following:

- *Track the target*: The tracking system must be performed in a reliable and close to real-time manner in order to avoid adding extra time, resulting in a delay, to the telerobotic task. Also, the ROI of the tracked object has to be well adjusted to the target contour in order to obtain better performance and accuracy. For this, it must be taken into account that the KCF algorithm is not invariant to scale. Therefore, when the camera approaches the lens, the ROI should be increased accordingly, avoiding losing the tracking that would otherwise occur. Likewise, when the camera is moving away from the target, the ROI has to be decreased, avoiding to track a wrong area, since the depth of the whole unstructured environment (where the robot is often used to perform the interventions) could generate errors. In summary, the tracking must be invariant to scale, orientation, translation, reflections due to metallic parts, lack of luminosity and partial occlusions.
- *Surround the target*: During intervention, according to the expert telerobotic human operators’ experience, it is very common to have to turn around the target once it is detected, due to the fact that the location of the components in an unstructured environment might need to dribble obstacles and study the best trajectory to reach the goal. Meanwhile, the tracking system has to be able to follow the ROI, helping to keep the target at the centre of the view.

The way to fulfil all the requirements listed above is to develop a system in which both KCF and a feature detector and extractor algorithm, work in a coordinated manner. In Reference [44] the most significant algorithms for that purpose were tested, the SURF [45] being the one that better suits our needs.

**Figure 3:**
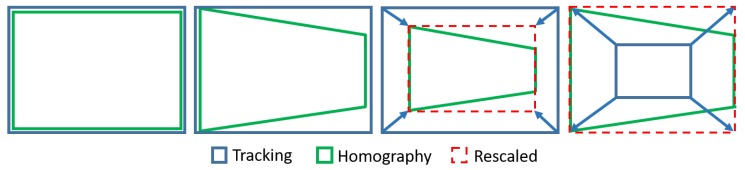
Left to right: Initial state; Rotation and/or translation in the same direction; Reduction of the region of interest (ROI) due to estrangement; Increased ROI due to the approach.

**Figure 4:**
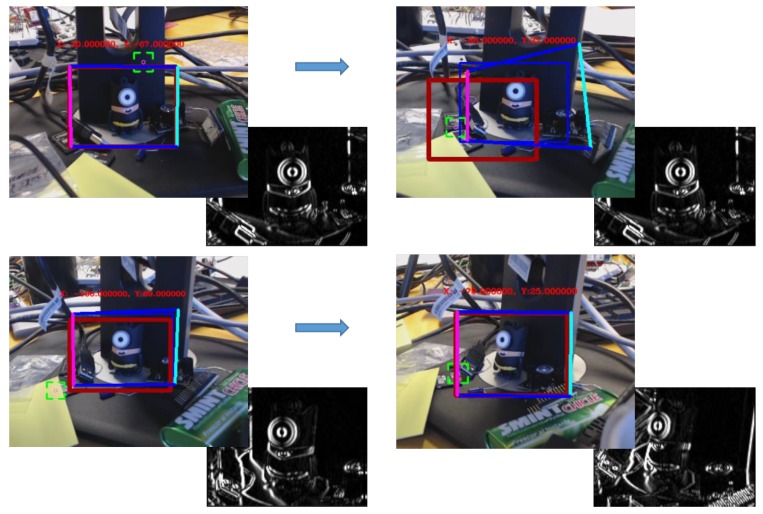
SURF+KCF system sequence working with different patterns in the same execution. The red square shows the position where the pattern was taken. The colourful square depicts the SURF-based homography estimation, and the blue square represents the tracked ROI.

The unified developed vision algorithm presents a greater tracking enhancement in terms of performance and accuracy. In fact, when KCF needs to adapt the ROI dimensions, this is rescaled by making use of the SURF-based homography estimation, by adjusting the ROI to the dimensions of the SURF bounding-box (see Figure 3). Instead, taking advantage of the fact that, broadly speaking, tracking algorithms try to follow what is being shown inside their ROI area frame by frame (unlike those feature extraction algorithms such as SIFT [46] or SURF among others, which focus on the search for an existing pattern), it allows to turn around the target without losing it. With that in mind, when SURF has problems to detect the target, KCF (that follows the object throughout the rotation and/or translation) provides a new pattern using the current ROI square, thus updating the existing one, allowing to the homography to improve its performance under the new visual orientation. As seen in Figure 4, the first two screenshots (both in the upper row) are sharing the same pattern and yet, the two in the second row use updated patterns, which have been obtained from the ROI’s covered area as soon as the homography begins to have issues with the area/object detection.

## 5. Tracking-Based Depth Estimation

The proposed solution to retrieve the depth estimation while tracking metallic objects must work close to RT with the aim of fulfilling the mission requirements. For this, aspects such as the visual operator feedback (critical to avoid the delay) and the data collection for both autonomous and semi-autonomous tasks must be taken into account. Besides, the robustness of the algorithm is a highly relevant key point, due to the fact that it is used in real robotic interventions on harsh and costly environments, where the safety of humans and scientific material is crucial.

**Figure 5:**
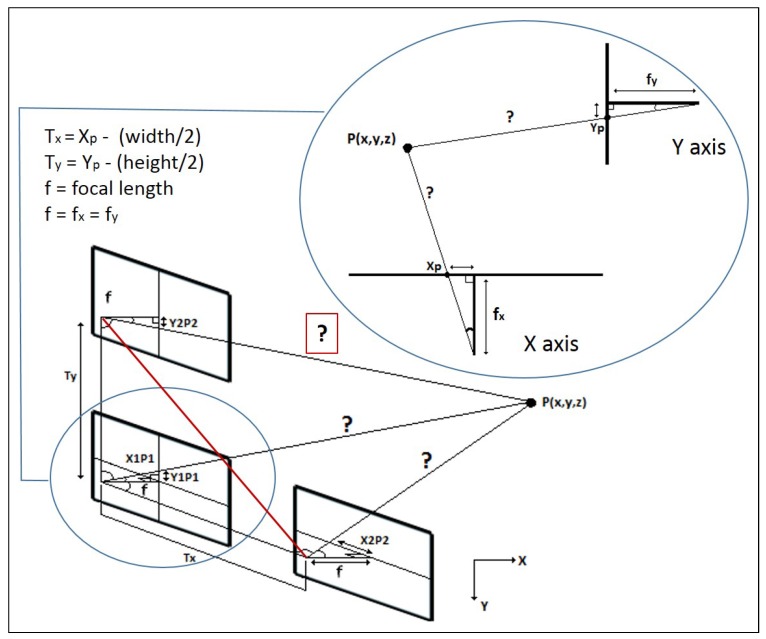
Triangulation proposal in *X* and *Y* axis. X1P1 and Y1P1 are the projection of the point (P) on the first image, and X2P2 and Y2P2 the projection of the same point on the second picture.

As a first step, the correlation between the key-points drawn from the pair of images has to be calculated, so as to triangulate the target position (see Figure 5), which will serve in order to: (1) adapt the robot velocity to the necessities with respect to the measured distance, and (2) to carry out the calculation of an adaptive trajectory to approach and reach the target, which is under development at the moment of writing.

For this purpose, it is mandatory to compute the camera world coordinate position at every time, which is achieved by means of the use of the forward kinematics [47] through at least 6 DoF robotic arms [48,49], which provides the current position of its end-effector (where the camera is attached) with regards to its frame (its base).

Hence, by applying a last homogeneous transformation to the matrix of the robot (as explained in Section 2.2), it is possible to get the exact position and orientation of the camera with regards to the robotic arm base, which leads to the transformation matrix calculation.

Once the system starts, the ROI of the first frame will be used as reference, and the second area of interest of the peer will be obtained from the current frame. For determining the correlation points set, the movement of the camera is calculated by the difference among the inverse of the initial homogeneous transformation matrix of the camera coupled to the end-effector (as explained above), and its current transformation matrix, getting the translations in *X* and *Y*-axis. In order to make balance on the correlation system regarding to the possible rotations done, Euler [50] is applied to get the angular of these from the Equation (3).

(3) $$M_{f}=M_{f}^{-1}\cdot M_{C}$$

Having calculated the correlation of the key-points inside of the ROIs, the Sinus Theorem (see Algorithm 1) is applied to achieve the triangulation for each key-point (for the purpose to extract their average, due to the fact that the piece/area might no be a flat surface), in which the estimation of the distance among the target and camera is based on the translation and rotation of the camera (see Equations (4)–(6)) where: $P_{1}$ and $P_{2}$ are the projection of the key-points on the origin and current picture respectively, as well as $hypo_{1}$ and $hypo_{2}$ are the hypotenuses for each image, both the focal length ($f_{x,y}$) and the center point (CP) come from the intrinsic parameters of the camera (see Equation (1)), and *T* (translation) and *R* (rotation, always the opposite to the translation) are the intrinsic parameters determined by applying the Equation (3).

**Algorithm 1** Depth estimation algorithm based on the tracking solution.**Require:**distanceX≥0.001 OR distanceY≥0.001 **function**
depthEstim($Transl_{x},Transl_{y},Rot_{x},Rot_{y}$)   **for**
square←0toNSquares
**do**    **if**
square is not outlier **then**      distance($Transl_{x},Rot_{x},Depth_{x}$)      distance($Transl_{y},Rot_{y},Depth_{y}$)    **end if**   **end for**   normalDistribution($Depth_{x},Depth_{y}$)   **if** depth is estimated **then**    Show the estimation and the error by AR and to adapt the arm velocity regarding to the depth   **end if** **end function**  **function**
distance(T,R,Depth)   $hypo_{1}=\sqrt{{\left(P_{1_{x,y}}-CP_{x,y}\right)}^{2}+{\left(f_{x,y}\right)}^{2}}$   $hypo_{2}=\sqrt{{\left(P_{2_{x,y}}-CP_{x,y}\right)}^{2}+{\left(f_{x,y}\right)}^{2}}$   **if**
$P_{1}<P_{2}$
**then**    $hypo_{1}\Leftrightarrow hypo_{2}$                     ▹ Swapping pictures position   **end if**   $\alpha_{x,y}=\pi /2-\arcsin\left(\left(P_{1_{x,y}}-CP_{x,y}\right)/hypo_{1}\right)$   $\beta_{x,y}=-\left(\pi /2-\arcsin\left(\left(P_{2_{x,y}}-CP\right)/hypo_{2}\right)+R_{x,y}\right)$   $\gamma_{x,y}=\pi -\alpha_{x,y}-\beta_{x,y}$   $Depth=\left(-T_{x,y}\sin\left(\alpha_{x,y}\right)\right)/\sin\left(\gamma_{x,y}\right)$ **end function**

(4) $$\alpha_{x,y}=\frac{\pi }{2}-\arcsin\frac{P_{1_{x,y}}-f_{x,y}}{hypo_{1}}$$

(5) $$\beta_{x,y}=-\left(\frac{\pi }{2}+R_{y,x}-\arcsin\left(\frac{P_{2_{x,y}}-f_{x,y}}{hypo_{2}}\right)\right)$$

(6) $$DE_{x,y}=-T_{x,y}\cdot \sin\left(\alpha_{x,y}\right)/\sin\left(\pi -\alpha_{x,y}-\beta_{x,y}\right)$$

To perform this estimation, it has been mandatory to carry out the camera calibration beforehand, by obtaining the focal length from its intrinsic parameters (explained on the intrinsic parameters layer of the Section 2.2), which is strictly required to display the key-points projection on the 2D plane that is generated by each image of the triangulation system (see Figure 5).

Considering that the system design is made to allow free movement in space, it does not need to know the original position of the system reference frame and the camera rotation in *X* and/or *Y* axes during the motions.

### 5.1. Metallic Pieces Detection

**Figure 6:**
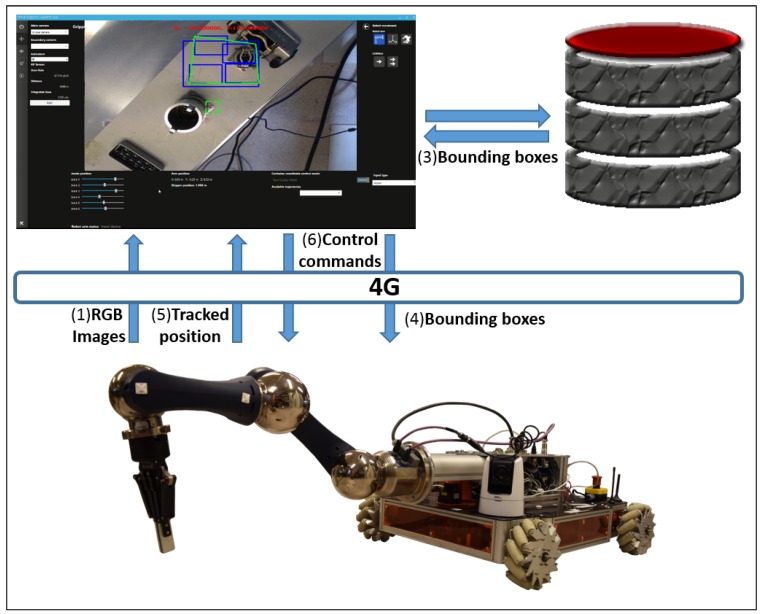
Deep Learning integration diagram.

With the aim of offering to the user a higher level of interaction with the system, a deep learning-based module for object recognition [51] has been integrated (see Figure 6), which allows metallic object recognition in a robust manner (e.g., connectors, sockets and patch panels) upon non-textured attributes. The module is based on Faster-RCNN already pre-trained in COCO [52].

The neuronal model that showed greater accuracy for this technique, by detecting a large number of metallic parts of our interest, is the ResNet-101 [53], which obtained total losses below 0.05%, better results than other models such as Inception-v2 [54] and ResNet-50, where the score was over 0.1%. In total, 500 pictures of 6 different objects of interest (see at Section 7.2) were used to train the method along 100,000 steps. However, the loss function already converges for classification and box estimation in step 30,000, by using the same COCO parameters. The performance of this solution is capable of detecting objects at the remote robotic site in under 1 s (network dependency), delivering a bounding box for each detected object to the HRI, which will be offered to the operator, allowing him to directly choose the object to be tracked, starting the depth estimation procedure.

Also, it is important to clarify that the metallic pieces detection using the neural network techniques is applied when required by the operator, normally when the target is faced to the robot and before the intervention starts. On the other hand, the tracking system is working continuously on the robot side. We cannot tell the system is working in real time because the 4G network that connects the robot to the surface is not providing this capability. Anyway, the system looks for the best performance in order to improve the efficiency of the intervention.

### 5.2. Features-Extractor-Based Key-Points Correlation

As a first approach, the system has been designed using the SURF-algorithm as a basis, which has been used to obtain an adequate set of key-points for finding the correlation between the origin and current images. The ensemble of features provided by SURF have to be treated appropriately in order to be useful to the next step of the algorithm.

Since the set of key-points are extracted from the ROI of the scene (reducing the computing time), these must be reallocated on the plan (looking for the real position upon the view) increasing *X* and *Y* with regards to the ROI origin coordinates.

**Figure 7:**
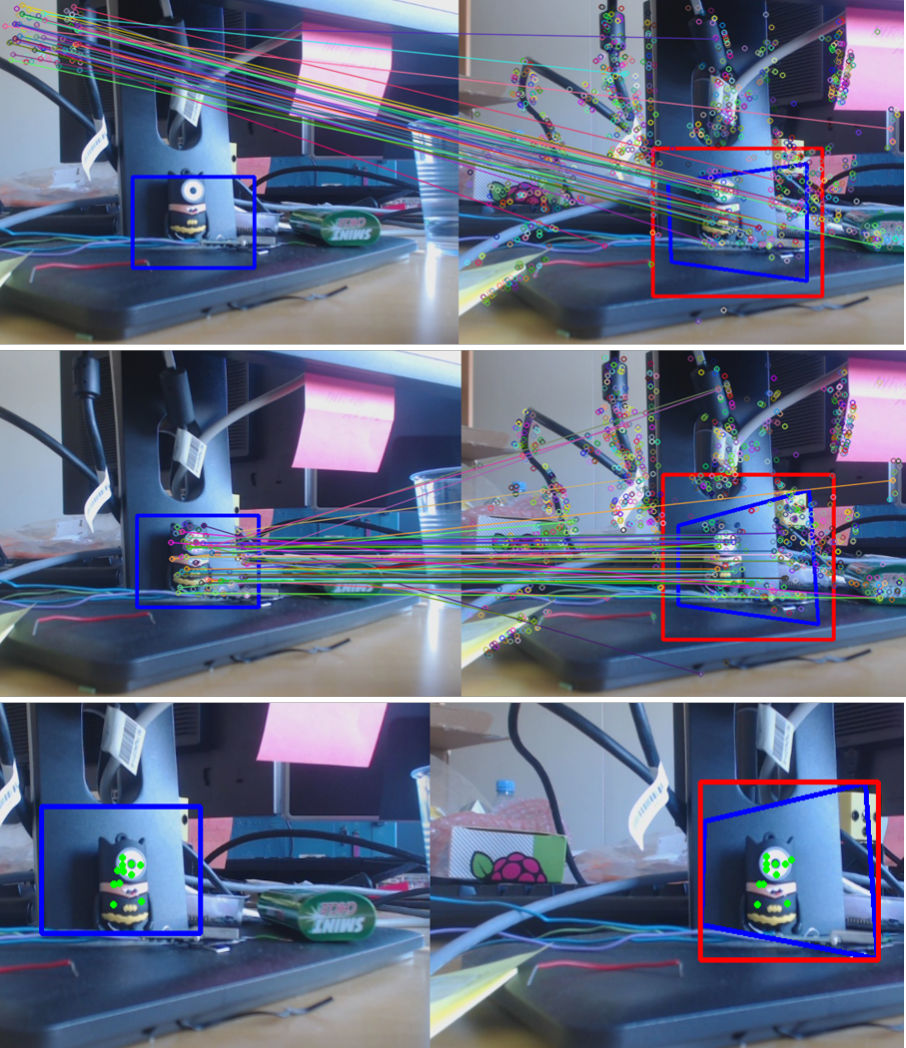
(**Top**) key-points correlation between two images in raw. (**Middle**) key-points correlation among two pictures after the isolated ROI (used as a pattern) translation adaptation. (**Bottom**) key-points correlations after filtering with the euclidean distance-based threshold.

Then, the outliers are filtered, which include both the points out of the bounding box, as well as those that, according to the Euclidean-distance [55], are greater than the experimental threshold established previously (see Figure 7).

In order to help the robot operator reach the proper position to perform the task, or to guide the mobile manipulator autonomously to attain the target, the system must send the robot’s next position meanwhile the estimation of the distance to the target is carried out. Because of the instability of the homography performed by SURF in these kinds of scenarios where the lack of features and metallic surfaces are the most common situation, KCF was used instead, with the goal of smoothing the current position of the interest area, tracking it and estimating the next position. The use of the vision system and depth estimation has also demonstrated the improvement the focus and quality feedback sensation of the operator, avoiding undesired cognitive fatigue.

Besides this, the robotic arm work-space and reach-ability [56,57,58] was considered in the algorithm, since the limits of the robot movements or the positions reached due to singularities [59,60] can affect directly the estimation, being necessary in those undesired situations to correct the arm position and restart the assessment.

### 5.3. Tracking-Based Key-Points Correlation

Despite the fact that SURF-based results show an excellent performance in terms of accuracy, they also show instability in the required scenarios, where the object and its surroundings are metallic, with very poor texture features, and the possibility of glares and partial occlusions. Due to this situation, it has been necessary to redesign and find out an extended solution to fix the weakness of the approach exposed above (Section 5.2).

KCF has been used as a replacement for SURF as key-points supplier with the aim of gaining this necessary stability, sacrificing such essential characteristics as the homography and the partial occlusions that feature extractor algorithms commonly offer, facilitating the correlation task.

**Figure 8:**
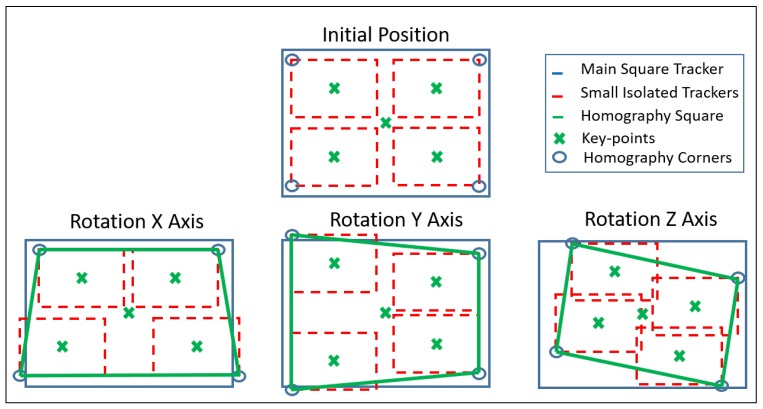
Rotation solution for the homography.

In order to take advantage of the enhancements made with the use of KCF and with the goal of overcoming the above-mentioned disadvantages, the algorithm proposed uses five tracking regions instead of one (see Figure 8). The main frame represents the whole ROI, which is divided into four mini-trackers, of which the centre will be considered as the key-points. Then, each little square will work independently, tracking its own area, correlating the key-points (within our selected screen region), between the points of the original and current images.

Because of this, the problems presented by the partially hidden targets and the constraints from the invariance to rotations are arranged, allowing full freedom movements to the camera, crucial to the proper system performance, which joins the properties of each algorithm, compensating the weakness of one another.

On the other hand, due to the featureless ROIs covered by the trackers, a Euclidean-distance-based threshold has been established (see Algorithm 2), which compares the behaviour of each tracking with each other, trying to predict the performance of each tracker. This allows the detection of the potential misbehaving of the squares. This guarantees the supply of a set of data with best key-points correlations, after filtering the outliers and avoiding the disturbance of the estimation made by the triangulation. The estimation can also be restarted if necessary.
**Algorithm 2** Euclidean-based threshold to avoid the wrong behaviour of the squares, where rRf is the initial reference position of each ROI, and thresholdErr the threshold set by the user. **function**
checkRoiStatus(rRf,thresholdErr)   **for**
ROI←0toN_ROIs
**do**    **if**
ROI is not outlier **then**      edp=calculate_eucliDisPoint[ROI,rRf[ROI]];      euclideanDistPoint.push_back(edp);      distance_average += euclideanDistPoint[ROI];    **end if**   **end for**   distance_average/=N_ROIs;   **for**
ROI←1toN_ROIs
**do**    error = (distance_average*(thresholdErr)/100)    **if**
euclideanDistPoint[ROI]>|error|
**then**      avoidRoiToDepthStimation[ROI]=true;    **end if**   **end for** **end function**

## 6. System Testing and Commissioning

Object tracking and depth estimation have been integrated and tested in both autonomous and supervised systems, which are already fully integrated into CERN’s robotic framework, endowing it with an artificial intelligence capable of guiding the operator (i.e., supervised performance) or performing the task by itself (i.e., autonomous behaviour).

The testing and commissioning on real interventions have been carried out under the use of different kind of industrial cameras, from normal webcams to endoscopic ones (see Table 1 in Section 1.2).

In fact, in this section the following use cases are going to be described: (1) example of vision-based autonomous behaviour, where the tracking and depth information is used to automatically perform an intervention in the panel of a machine present at the LHC; (2) example of a semi-autonomous vision human-supervised task, where the operator uses the vision system to assist in an intervention task with a connector; and (3) the contingency behaviours added to the vision algorithm to enhance its safety and accuracy for real interventions.

### 6.1. Example of Vision-Based Autonomous Behaviour

**Figure 9:**
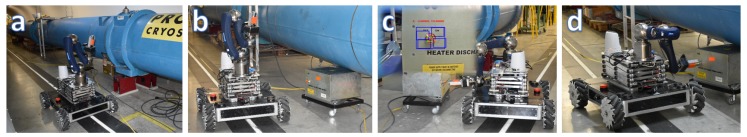
Task sequence carried out by CERNBot2: (**a**) target detection through a Pan–Tilt–Zoom (PTZ) camera, (**b**) approaching to the target, (**c**) depth estimation (**d**) switch actuation.

**Figure 10:**
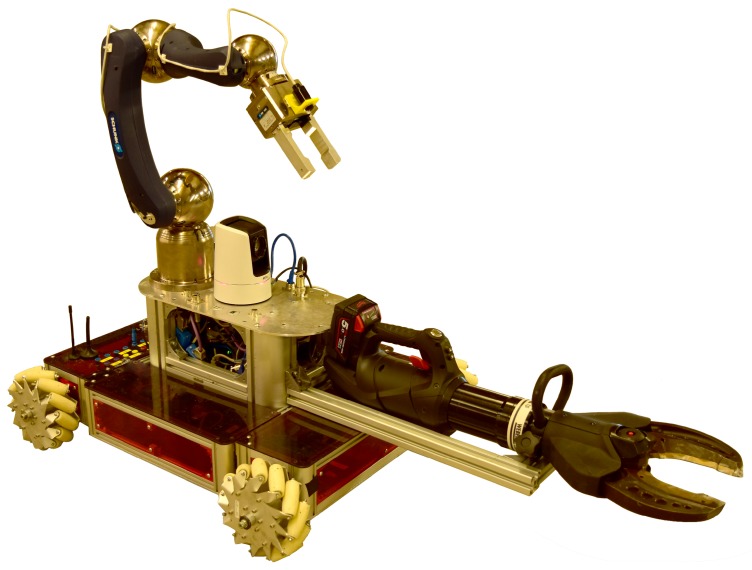
CERNBot in scorpion setup. Here it can be seen how the robot wears the two kind of Axis cameras outline above.

**Figure 11:**
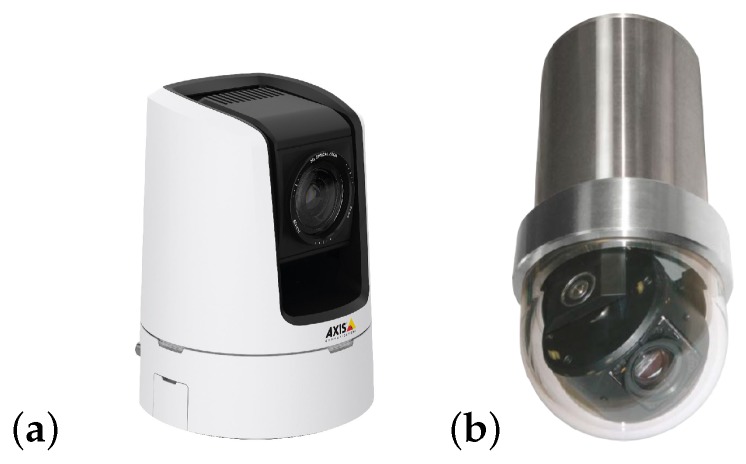
Cameras PTZ: (**a**) Axis V5914 PTZ, usually attached on the CERNBot platform. (**b**) Bowtech BP-DTR-100-Z underwater camera for further use in radioactive dust and underwater scenarios.

**Figure 12:**
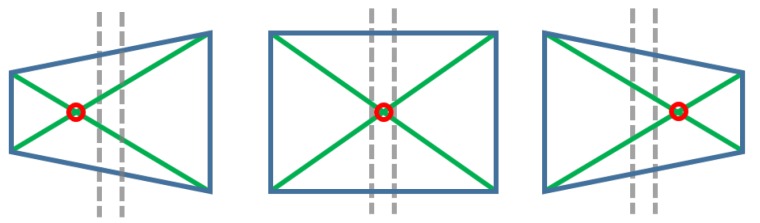
Use of the square-homography intersection to fix the orientation. The squares meaning is: the left one needs to turn right, the one at the centre is well oriented, the right one needs to turn left.

This system is a state machine developed with the aim of detecting a switch on a Heater Discharge Power Supply (QPS) to turn it on/off, which is largely present at the LHC tunnel. The system has shown very good results, being a nice example of how powerful the ecosystem created by SURF+KCF can become (see Figure 9). The state machine is composed by the following states:

- *PTZ-Camera Visual Servoing Robot Control*: A visual servoing system has been deployed to drive the robot (see Figure 10) to the target through an Axis PTZ-camera (see Figure 11), which is in charge of finding out the QPS, making use of the SURF algorithm, and a set of patterns previously loaded. Due to the fact that the camera’s framework uses the internet network protocols, a request and response communication-based Python controller has been embedded on the system (see Listing 1) to guide the platform and position it in front of the target in a proper distance, so that this can be reached.
- *Vision control for arm orientation*: With the robot arm already approaching the target, the robotic arm is triggered to a specific position and the gripper camera is switched on, while the PTZ-camera remains disabled. Thus, the orientation of the robot with regards to the target device is calculated by the homography provided by SURF through the gripper camera. For that purpose, the intersection of the opposite corners of the square-homography gives the current orientation, as seen in Figure 12.
Listing 1: Request & response for PTZ-camera control via Ethernet network using Python and CURLib in parallel.#include <python2.7/Python.h> #include <curl/curl.h> .... // It launches a request to the camera and returns its orientation int queryPositionPTZCam(std::string &queryAxis) { std::string readBuffer; CURLcode res; CURL *curl = curl_easy_init(); if (curl) { curl_easy_setopt(curl, CURLOPT_URL, queryAxis.c_str()); curl_easy_setopt(curl, CURLOPT_WRITEFUNCTION, WriteCallback); curl_easy_setopt(curl, CURLOPT_WRITEDATA, &readBuffer); res = curl_easy_perform(curl); curl_easy_cleanup(curl); } // Extracting the orientation figure from the response received return std::stoi(readBuffer.substr(readBuffer.find("=") + 1, readBuffer.find("\n") - readBuffer.find("=") - 1)); } void movingCamera(const cv::Point centerScreenPoint, cv::Point &centerError) { // If target position is reached, stop PTZ-Camera if (queryPositionPTZCam(queryAxis) == targetPosition) goto stopCamera; Py_Initialize();  // Instantiate a Python’s interpreter // Lambda function, it executes Python’s instructions within the // scope of the interpreter auto sendRequest = [&](std::string &URLRequestAxis) -> void { PyRun_SimpleString("import requests"); PyRun_SimpleString(URLRequestAxis.c_str()); }; // If the target is not center in the scene if ((abs(centerError.x) > 1 or abs(centerError.y) > 1)) { sendRequest(movementURLPythonRequestAxis); } else if ((abs(centerError.x) <= 1 and abs(centerError.y) <= 1)) { stopCamera: sendRequest(stopURLPythonRequestAxis); } Py_Finalize();  // Closing the interpreter }
- *Depth Estimation*: insofar as the switch detection is done (by using the split left side of the frame, since the switch location is perfectly known), the depth estimation presented in this document is launched, providing the distance to the camera and placing the gripper towards the switch.
- *Fine-grained approach to the target*: Apply $T_{Z}$ (see Equation (7)) translation upon approaching direction by inverse kinematics to reach the switch, where $d_{Z}{}_{e}^{c}$ is the distance from the camera to the end-effector, taken from the *approach* parameter on Equation (8) (wherefore $M_{c}^{f}$ and $M_{e}^{f}$ are the position of the camera and the end-effector respectively with regards to the TCP), and *Depth* is the measure done by *Depth Estimation* algorithm. The velocity is adapted to the distance estimated for a proper performance.
  (7) $$T_{Z}=\left|\begin{array}{cccc} 1 & 0 & 0 & 0\\ 0 & 1 & 0 & 0\\ 0 & 0 & 1 & Depth-|d_{Z}{}_{e}^{c}|\\ 0 & 0 & 0 & 1 \end{array}\right|$$
  (8) $$d_{Z}{}_{e}^{c}\in \left({M_{c}^{f}}^{-1}\cdot M_{e}^{f}\right)$$

### 6.2. Example of Semi-Autonomous Vision Human-Supervised Task

Taking into consideration that the vision system can work autonomously in controlled environments, it is worth mentioning that, in order to perform such an intervention on unstructured hazardous environments and expensive scientific facilities, it is necessary to keep an operator always in the loop, which can supervise the semi-autonomous behaviours, stop them if necessary and even take manual control of the robots due to unexpected situations.

**Figure 13:**
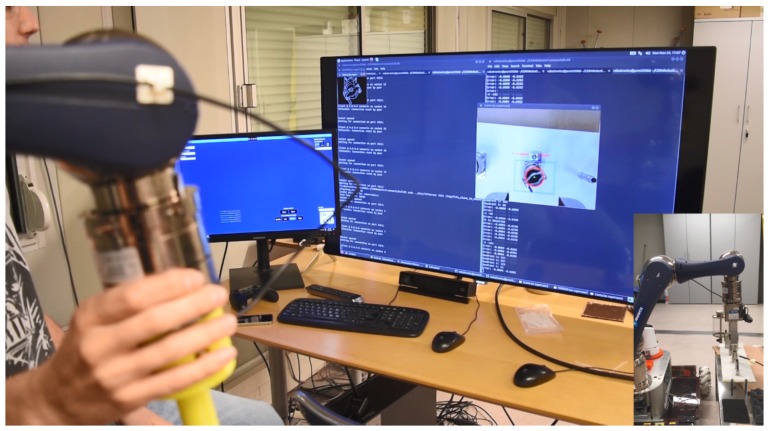
Operator guidance by tracking-based Depth Estimation system upon metallic surface.

The proposed human-supervised control solution has been integrated in the CRF, including both the server controller and the client Human-Robot Interface. The CRF gets the required ROI from the HRI, as explained in Section 5, either from an area of interest selected by the operator, or running some object detection solutions, after the required training and setup. Then, the HRI will show to the operator the sensors feedback provided from the system, through a multimodal and augmented reality module and it shall adapt the robot velocity to the perceived depth. Besides this, if the operator considers it necessary and safe, the automatic tracking can take control of the arm to approach the target in an smooth manner, trying to avoid mistakes on the approaching time and keeping the goal centred in regards to the frame (see Figure 13).

It is worth noting that the visual feedback to the operator runs according to the frame rate of the camera used, since the frame shown on the GUI shall be the current one, although the information goes relative to computational load, avoiding bad sensations to the operator as well as dealing with the possible tiredness.

### 6.3. Contingency Behaviours

**Figure 14:**
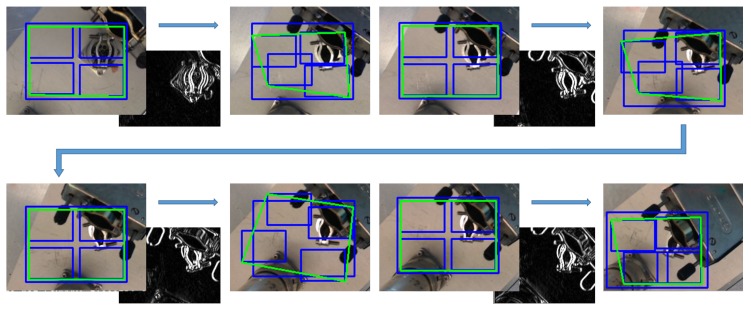
System behavior under partial occlusions.

**Figure 15:**
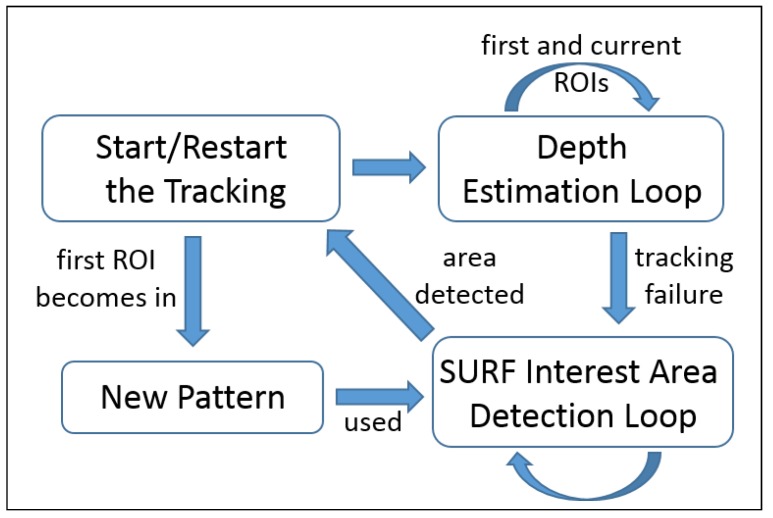
Recovering System diagram.

Bearing in mind the robustness and stability that the system must show working in costly and unstructured scenarios, the contingency plans become primordial by anticipating the possible losses of the targets generated by the uncontrollable situations (i.e., full target occlusion, ROI disappearance due to the robotic platform has gone through an obstacle/crack, etc.). If so, the estimation and guidance stop, switching on SURF (which will deal with the retrieval of the tracker) making use of a pattern that was previously extracted from the main ROI, which can be traced back from Figure 14 (binary pictures) when the tracking updates its situation. Meanwhile, the robot gets stabilised afterwards in order to get out from the crack (or the operator turns the view to the target again) and the system looks for the reference in the scene, which shall allow the continuation of the estimation and the guidance that was being carried out. Hence, applying a feature extractor (such as SIFT or SURF) collaboratively with the enhanced tracker exposed on the document, generates a greater robust ecosystem, able to deal with undesired situations as well as to self-heal from the issues (Figure 15).

## 7. Results

### 7.1. Accuracy Experiments

**Figure 16:**
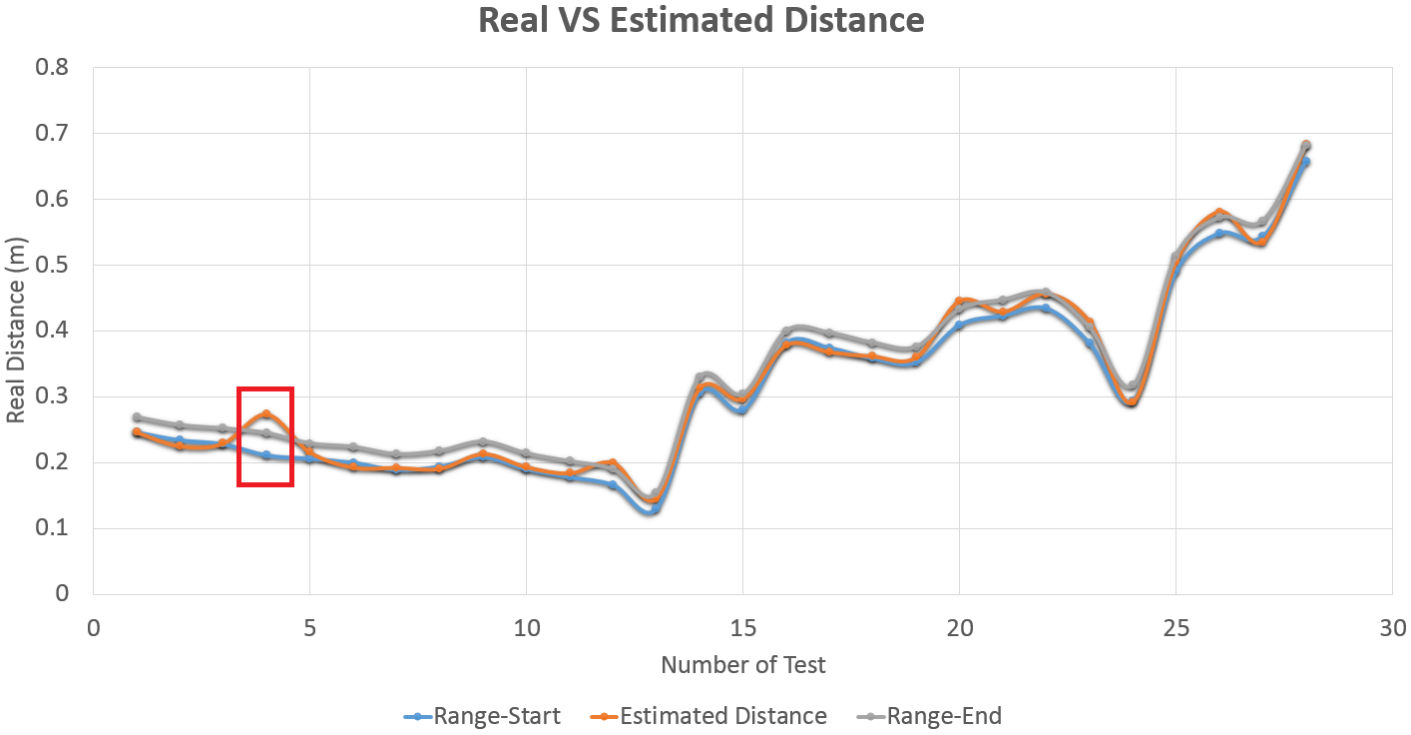
Confidence test. Grey and blue lines represent the current distance range. Orange line is the estimation. Red box shows the only one error over 1 cm.

The presented module, which is currently being used in real robotic interventions within CERN’s facilities in a successful and robust manner, is capable to run in both harsh and featureless environments, providing guidance and surrounding (either for a robotic platform as for the operator in charge), and a depth estimation by correlating a set of key points in a novel way and be close to RT performance. The system that is integrated in the CERN’s HRI, achieves an accuracy of over 90% in the depth estimation measurements (see Figure 16), with under one centimetre of error.

**Figure 17:**
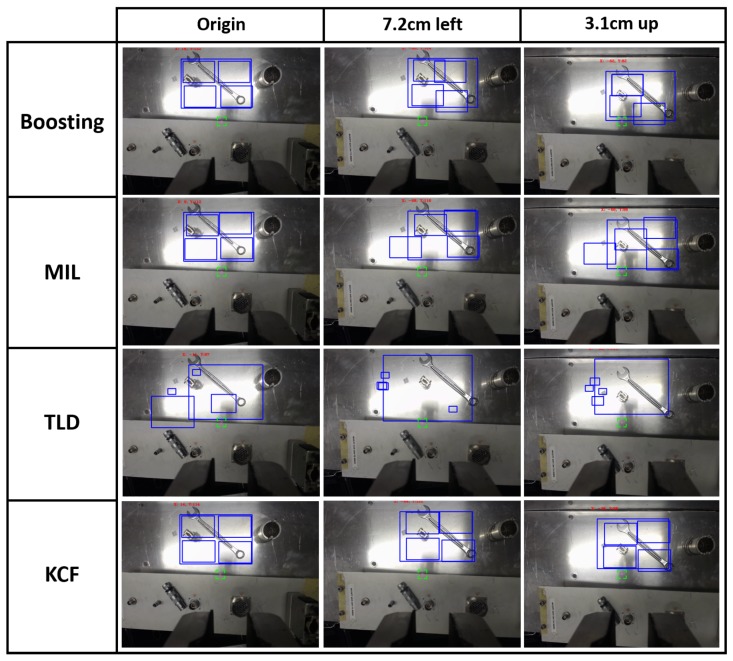
The performance of monitoring algorithms tested prior to system development, where it is shown the origin position, the translation in *X*, and translation in *Y*. Translations are with respect to the robot TCP.

With these matters, the set of current tracking algorithms tested and described above (see Section 2.1) has been integrated into the system developed to prove its performance, and thus demonstrate why all those whose properties do not provide what is necessary for the development of our tracking system have been rejected. Therefore, as seen in Figure 17, KCF showed the best performance in consideration of both computational time and accuracy.

**Figure 18:**
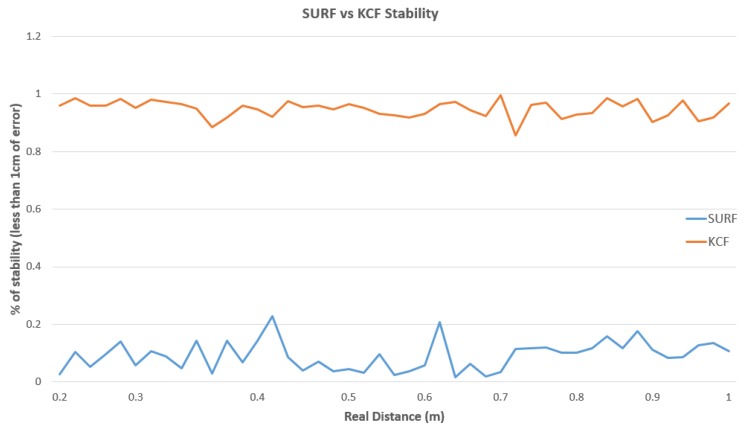
Difference of stability in the performance of the two solutions proposed (SURF-based and KCF-based), where the abscissa axis represents the distance to the target and the ordinate axis the percentage of frames providing the correct measurement.

Figure 18 shows the yield of the novel algorithm that is proposed in this document, where the depth estimated by SURF and KCF solutions are compared against known distances, which present large differences in terms of stability. Regardless of the high accuracy that both solutions have proved (see Table 3) with an average error under 0.5 cm, is the KCF-based one that represents the higher level of stability, achieving more than 94%. This percentage comes from the amount of frames that provides the correct measurement. Once the distance is achieved, it keeps the camera in the final position, removing the frame outliers per thousand samples.

Furthermore, joining the opposite corners from the isolated little squares, the system overcomes to the well-known rotations constraints that the tracking algorithms show (see Figure 8 in Section 5.3) and the partial occlusions (see Figure 14) that could happen in the time frame that the robot moves carrying out the interventions, besides allowing the possibility to calculate the homography for those systems with missing matrix of the robot.

**Figure 19:**
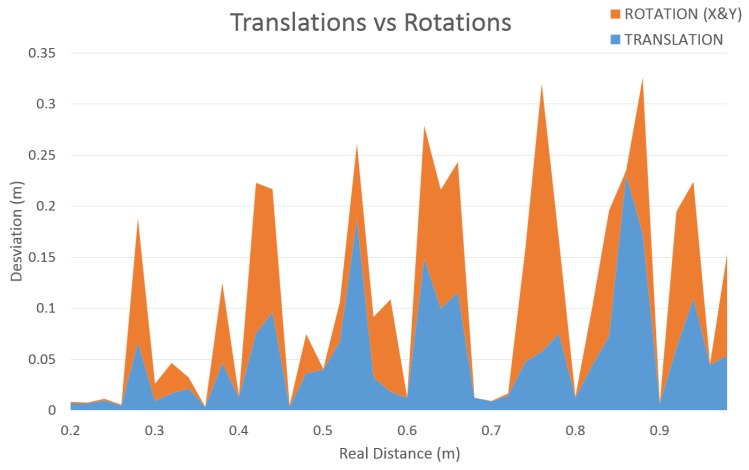
Relationship among translation and rotation (*X* and/or *Y* axes) to achieve the triangulation.

In addition, the algorithm endows maximum freedom of movement to the robotic arm, translating and rotating the camera either in *X* and *Y*-axis. Due to this, the translation must be increased in regards to the rotation done (see Figure 19), compensating it and avoiding the drop of the set of key-points outside of the scene or the exchange of their position within the triangulation system.

### 7.2. Metallic Targets Data-Set for Tracking and Object Recognition Benchmarking

In order to enable further tracking and object recognition experimentation on metallic targets, the used data-set is provided, which is available at (https://cernbox.cern.ch/index.php/s/08vGzLeQ1w9CFac).

In Table 4 the list of objects that have been used to train the object recognition neural network can be found.

### 7.3. Videos

Some videos of the experimentation are also provided in this section.

- Contingency behaviour: this video shows a safety contingency procedure used in the tracking and depth estimation algorithm, to avoid the robot to move once the tracking has been lost, and also helping it to recover the track once the object is facing the camera. For this, once the tracked object is lost, the last tracked ROI is used by a tracking thread to explore the next camera frames, which allows the system to better recover the track according to the new reflections and luminosity target state. (https://cernbox.cern.ch/index.php/s/kEIIK6hdPwnUdDk)
- Depth Estimation: in this video a robotic arm with on-hand camera facing a pool of metallic connectors (i.e., targets) is presented. First of all, the video shows the selection of the ROI by the operator, which enables the tracking and depth estimation procedure. Also, in the second part of the video the connectors are recognized by the deep learning algorithm. Then, once the operator selects to object to track, the system calculates its depth. (https://cernbox.cern.ch/index.php/s/Qguw2RMNLr0SwuO)

## 8. Conclusions and Future Work

**Figure 20:**
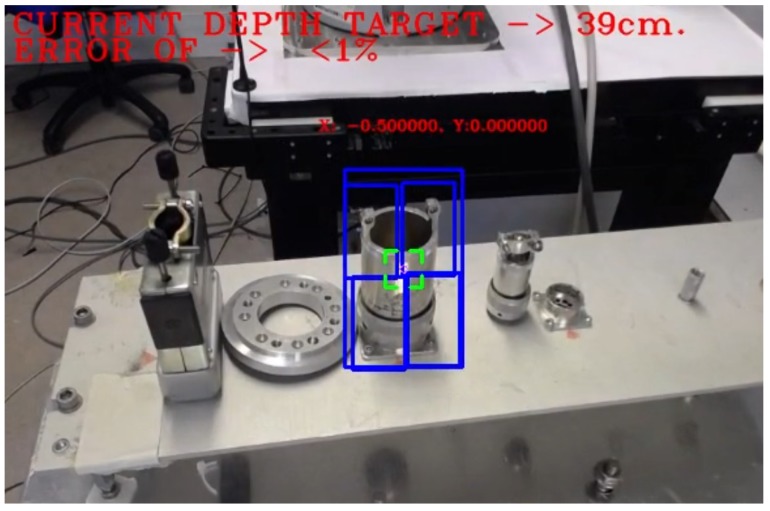
System execution, where the tracking and estimation is shown by AR.

This paper has presented a tracking based depth estimation system including the recognition of metallic objects, which has been successfully developed (see Figure 20) and validated at CERN to perform real telerobotic interventions in radioactive environments. The system permits the calculation of the depth at which a metallic target is located, once this has been detected by either an operator selection, or using a deep learning algorithm, with the aim of assisting the expert operator during human-supervisory control of the robot platform, including semi-autonomous vision-based interventions. The information obtained from the vision system is represented in the HRI in a multimodal and augmented reality manner. Due to the necessity of using the system in real interventions in the LHC tunnel, which is a huge responsability in terms of equipment where the operation has to be accomplished, the priority of the system is to guarantee the safety, while providing efficiency and reliability. For this, the vision system needs to work appropriately in the presence of reflections, light constraints and in partially occluded scenarios.

**Figure 21:**
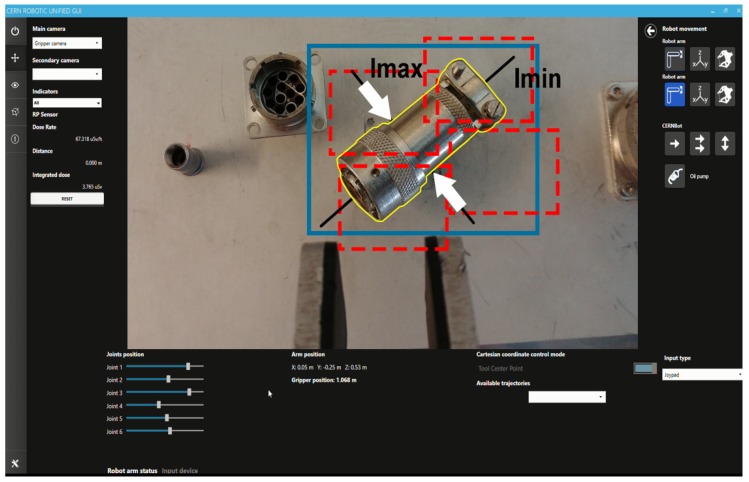
Grasping Determination calculation on a metallic connector, to be grasped and inserted (specular symmetry).

**Figure 22:**
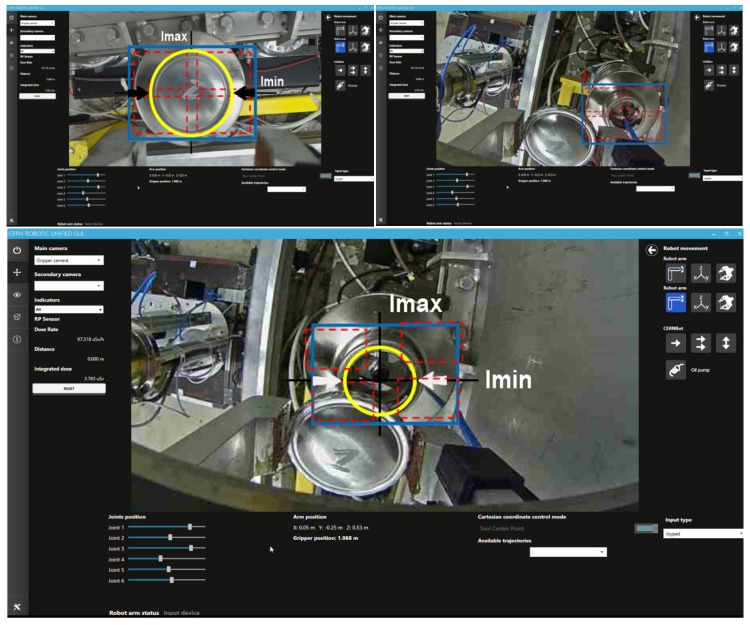
Grasping Determination calculation on a rounded and symmetric metallic object (radial symmetry).

As shown in Figure 21 and Figure 22, current and further work will focus on enhancing the vision system in order to calculate the grasping determination of the target in a fast and reliable way.

Having this objective in mind, the contour extracted from the tracking and object recognition algorithm is going to be processed by calculating the list of stable grasping points by using the algorithm explained in Reference [61], according to the symmetry knowledge.

The 2D grasping determination can also be adapted in 3D using a more sophisticated extension of the algorithm, as explained in Reference [62].

## Figures and Tables

**Table 1:** Specifications of the Used Cameras for System Validation.

Image	Model	Description
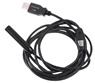	Endoscope VOLTCRAFT BS-24	HD 720p, 54°field of view, IP67-rated, diameter 8 mm., length 50 mm., 4 white LEDs light source, USB 2.0 connection
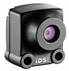	iDS uEye XS	HDTV 720p, 8MP CMOS sensor, dimensions: 23 × 26.5 × 21.5 mm., 12 grams weight, USB 2.0 connection
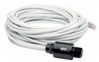	Axis F1005-E	Full HD 1080p, 113°field of view, IP66-rated, Ethernet connection
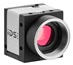	iDS uEye-se	VGA to 10.5 MP, CMOS sensor, up to 93 fps in AOI mode, USB 2.0 connection
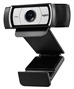	Logitech C930e	Full HD 1080p, 90°field of view, Auto-focus, zoom to 4X in 1080p, USB 3.0 connection

**Table 2:** Examples of grippers set and configuration of the cameras.

Setup	Description
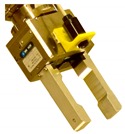	Default CERNBot’s end-effector with 7 cm fingers length and eye-in-hand mono-camera attached to the Schunk GP15 gripper
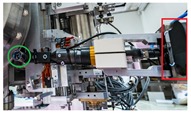	2 monocular cameras TCP system: red box shows an eye-in-hand camera; green circle shows an end-effector endoscope camera on the pneumatic angular screwdriver key held by the Schunk GP15 gripper
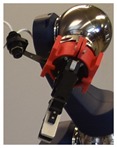	Axis monocular camera attached to a ROBOTIQ 2-Finger 140 mm Adaptive gripper
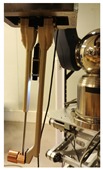	Endoscope eye-in-hand camera on the Schunk GP15 gripper with extension fingers (22 cm. length) for fragile and hardly reachable radioactive source manipulation
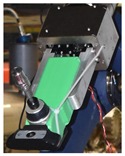	Webcam attached to the screwdriver head and carried by a Schunk GP15 gripper

**Table 3:** Stability and accuracy from both solutions presented.

Real Dist.	SURF-Error (m)	KCF-Error (m)	SURF-Stability (%)	KCF-Stability (%)
0.20 m.	0.0022346	0.0047224	0.083957663	0.970001464
0.30 m.	0.0046036	0.0043484	0.088593119	0.963883933
0.40 m.	0.0020228	0.0055516	0.12287338	0.92588872
0.50 m.	0.0038266	0.0033858	0.055642677	0.959756087
0.60 m.	0.0033424	0.0044288	0.049432454	0.932125796
0.70 m.	0.003555	0.0051822	0.068023731	0.959685824
0.80 m.	0.0041976	0.0060142	0.111163444	0.926650095
0.90 m.	0.002221	0.0045714	0.1359251	0.952898557
1.00 m.	0.0020276	0.0026512	0.108168958	0.939239064
Average	0.00311458	0.004539556	0.09153117	0.947792171

**Table 4:** Summary of the Data-set used to train the metallic targets object recognition module.

Image	Metallic Object	Description
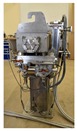	Collimator	Device present in the LHC accelerator to filter particles that got not aligned in the beam
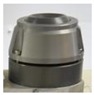	Guide	Beacons for alignment used in different tasks according to the ending placed on top
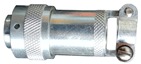	Socket	Electrical socket connection
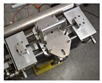	Separator	Device to separate the collimator from the beam
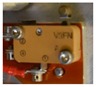	Relay	Switch to turn off/on the machine functions
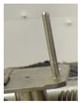	Spikes	Guide designed to help the the separator installation

## Abbreviations

The following abbreviations are used in this manuscript:

CERN	European Organization for Nuclear Research
LHC	Large Hadron Collider
BLM	Bean Loss Monitor
HRI	Human-Robot Interface
ToF	Time-of-Flight
RT	Real-Time
HD	High Definition
PTZ	Pan–Tilt–Zoom
MIL	Multiple Instance Learning
TLD	Tracking-Learning-Detection
KCF	Kernelized Correlation Filters
HOG	Histogram of Oriented Gradients
ROI	Region of Interest
RCNN	Regions with Convolutional Neural Network
TCP	Tool Center Point
SURF	Speeded-Up Robust Features
SIFT	Scale Invariant Feature Transform
DoF	Degrees of Freedom
QPS	Heater Discharge Power Supply
CRF	CERN Robotic Framework
GUI	Graphical User Interface
